# Local Anesthetics

**Published:** 1900-10-15

**Authors:** A. E. Mann


### LOCAL ANESTHETICS.

BY DR. A. E. MANN.

From an article on Anesthetics in Dentistry, published in the August *Ohio State Journal*.

In the use of topical agents hypodermically great care and judgment are required of the operator. The field of local anesthesia has been fertile in disappointments both to operator and patient. Although grave symptoms will sometimes follow even their most careful use, they have become a necessary adjunct to every dentist. Much of the ill effects follows their careless and indiscriminate use.

The vaso-constrictive power of cocain, together with the fear of the operation on the part of the patient, will often cause a cerebral anemia, which is the cause of the slight or grave symptoms that sometimes follow its use. Weak or very nervous patients especially require care, and if it is very warm or very cold weather, I prefer to have my patient in as near a horizontal position as possible during and for a few minutes after each operation. Personally, I

never inject more than one tooth at a time, and in this manner I feel my patient, so to speak. After extracting this one I wait from ten to fifteen minutes, and then if no toxic symptoms arise, I inject three to five more, and wait again if more teeth are to be extracted.

The strength and freshness of cocain solutions are of the greatest importance. As cocain is not very diffusible, a weaker solution will allow of a wider spread anesthetized field than a stronger solution.

To have uniform success one must not only have a proper strength solution but must know how to use it under the various circumstances, and in all pathological conditions of the gums. A 1 percent solution is the proper strength for healthy gums. For soft or spongy gums a 4 percent solution should be used. Make one deep injection on each side of tooth of about two minims each and extract almost immediately so as to get the parts to bleeding. By keeping quarter-grain tablets on hand the operator can make his solutions fresh for each case. Experience has proven that the addition of other drugs to the solution only tend to act as irritants and cause sloughing.

If the operation is of any magnitude it is well to have it follow a light but refreshing meal of which coffee is a part, and no dentist should administer this or any anesthetic without that which will antidote its toxic effects always on hand.

Of the syringe I wish only to speak of the needle. It should be neither too coarse or too fine; No. 23 Standard wire gauge is proper. It should be so ground that the planed surface at the end is about one-sixteenth of an inch long and round at extreme end instead of a fine attenuated point. This is important as the fine point is apt to separate the tissues instead of cutting them and thus hindering the absorption of the drug. The needle can in 95 percent of the cases be inserted painlessly in the following manner:

First assure patient that it will be painless, then place the planed surface on gums flat and press on the piston until you see some of the solution oozing around the needle; wait an instant, and then gradually work under the mucous membrane, all the time forcing the solution ahead of the needle; continue in this manner until the needle is in as far as you wish, which should be nearly opposite the end of the root and as near the edge of alveolus as possible. Buccaly or labialy the deep injection should carry enough of the solution to form a sack about the size of a split pea; the other should be near the gingival margin and at right angles to the root. This and the lingual or palatial injection should carry enough to whiten the tissues. The 1 percent solution will require from four to five minutes to anesthetize the parts.

While cocain is a dangerous drug, its intelligent use is a great boon to both operator and patient, as many people dislike the idea of being unconscious as under a general anesthetic.

Of more recent discovery we have the synthetical products, eucain and nirvanin. It is claimed for them that they are about five times less toxic than cocain and give an analgesia of longer duration. Also that their solutions can be boiled and are permanent, having a moderate anti bacterial action.

Both of these agents are rapidly coming to the front. This is due principally to the fact of their being less toxic than cocain. However, they are not entirely free from danger and the same care should be observed in their use as with cocain. A 5 percent solution has been found to give the best results with either. Eucain " B " is the salt recommended by the expert chemists, Messrs. Schering and Glatz.

A harmless and painless edema will sometimes follow their use, but it will seldom last more than one day. I

have found nirvanin to be even safer than eucain "B," and where I do not wish to use cocain I prefer this.

Orthoform as an anesthetic is very valuable for pain after extracting, by dusting the powder into the sockets; for buccal ulcers, burns or other sores about the mouth it is very beneficial in relieving the pain, as its anesthetic effect is of several hours' duration. Severe toothache, when due to an exposed pulp, I have found to be very quickly relieved by it. Used in pyorrhea pockets it enables the operator to scale the roots painlessly. Orthoform is also antiseptic and styptic.

Anesthetics have recently come into use very extensively in pulp removal, the method being as follows:

Take a very small piece of punk and saturate with a solution containing one part of formalin and four parts of alcohol; now place this on some powdered cocain crystals and place in the cavity and cover with unvulcanized rubber; then gently press on the rubber with a ball burnisher and in a few minutes you will have the pulp anesthetized. If pulp was not actually exposed you may have to make another application after removing the softened dentin. I have had almost uniform success with this method. Anesthetics are now also used extensively to obtund sensitive dentin.

The method of "Pressure Anesthesia," as suggested by Dr. W J. Morton, is a great stride in this direction. Pottassocain and vapocain, etherial solutions of cocain, when given sufficient time to act and properly covered in the cavity with beeswax, will in many cases almost entirely relieve the pain of drilling, and in most cases will lessen the pain somewhat.

It is a well-known fact that even a slight acidity of cocain solutions partially inhibits its anesthetic effect and that a slight alkalinity enhances it. For this reason pottassocain which is slightly alkaline, owing to the potassium contained, gives better results than vapocain. This has been my experience at least.

Whoever devises a method of forcing cocain into the dentinal tubuli that will work uniformly and not devitalize the pulp, will have accomplished this much-desired end.

Of ethyl chloride I will only say a word. With the other methods of producing analgesia, I see no use for its painful freezing spray, except possibly where you wish to remove a live pulp from a sound tooth or lance an abscess. I have removed the pulp of a sound tooth successfully, however, by injecting the 1 percent solution of cocain into the gums as near the apex of root as possible. In removing a live pulp from a sound tooth, or in lancing a painful abscess, the administration of the gas will often be very much appreciated by our patients.

